# Prevention, Assessment, and Management of Malnutrition in Older Adults with Early Stages of Cognitive Disorders

**DOI:** 10.3390/nu16111566

**Published:** 2024-05-22

**Authors:** Irene Loda, Emanuela D’Angelo, Emanuele Marzetti, Hanna Kerminen

**Affiliations:** 1 Scuola di Specialità in Geriatria, Università degli Studi di Brescia, Viale Europa 11, 25123 Brescia, Italy; i.loda@unibs.it; 2 Fondazione Policlinico Universitario “Agostino Gemelli” IRCCS, Largo A. Gemelli 8, 00168 Rome, Italy; 3 Department of Geriatrics, Orthopedics and Rheumatology, Università Cattolica del Sacro Cuore, Largo F. Vito 1, 00168 Rome, Italy; hanna.kerminen@tuni.fi; 4 Faculty of Medicine and Health Technology, The Gerontology Research Center (GEREC), Tampere University, Arvo Ylpön katu 34, 33520 Tampere, Finland

**Keywords:** aged, cognitive decline, DASH, healthy diet, malnourishment, Mediterranean diet, mild dementia, oral nutritional supplements, vitamin B, vitamin D

## Abstract

Malnutrition is common in older adults, and its risk is greater in those living with dementia. Relative to cognitively healthy peers, the prevalence of malnutrition is also increased in individuals with early stages of cognitive disorders owing to pathophysiological, cognitive, and psychosocial changes related to cognitive impairment. Malnutrition is associated with adverse health outcomes, including faster cognitive and functional decline. Here, we provide an overview of the prevention, assessment, and management of malnutrition in older adults, with a special focus on the aspects that are important to consider in individuals with early stages of cognitive disorders. Strategies to prevent malnutrition include systematic screening for malnourishment using validated tools to detect those at risk. If the screening reveals an increased risk of malnutrition, a detailed assessment including the individual’s nutritional, medical, and functional status as well as dietary intake should be performed. The management of malnutrition in the early stages of cognitive disorders should be based on the findings of a comprehensive assessment and be personalized according to the individual’s specific characteristics. In the article, we also provide an overview of the evidence on vitamin supplements and specific dietary patterns to prevent cognitive decline or attenuate its progression.

## 1. Introduction

The European Society of Clinical Nutrition and Metabolism (ESPEN) has defined malnutrition as “a state resulting from a lack of intake or uptake of nutrition that leads to altered body composition (decreased fat-free mass) and body cell mass, leading to diminished physical and mental function and impaired clinical outcome from disease” [1]. Approximately 20% of European adults aged ≥ 65 years are malnourished or at risk of malnutrition, but the prevalence of this condition varies greatly across communities and healthcare settings [2]. Malnutrition at an advanced age is associated with reduced quality of life, increased morbidity, hospitalizations, institutionalization, and mortality, as well as higher health care costs [3]. This highlights the importance of its prevention, early detection, and treatment.

Older adults are vulnerable to malnutrition due to multiple factors. Biological aging causes physiological changes in body functions that diminish the reserve capacities of organ systems and predispose to the development of malnutrition, especially in stressful situations [3]. Aging is also associated with a greater susceptibility to acute and chronic diseases [4]. Any acute or chronic disease has the potential to result in or aggravate malnutrition, as it frequently reduces appetite and food intake, impacts nutrient absorption and metabolism, and alters energy expenditure [3,5]. Therefore, malnutrition is especially prevalent in older adults with acute or chronic diseases, frailty, and functional decline [6]. 

Cognitive disorders and dementia are examples of chronic diseases associated with an increased risk of malnutrition [7]. Neurocognitive disorders are acquired conditions that involve deficits in neurocognitive functioning, including, for instance, memory, language skills, attention, executive functions, processing speed, and visual spatial skills. Cognitive impairment that is severe enough to interfere with an individual’s performance in everyday life is referred to as dementia. According to the International Classification of Diseases 11 (ICD-11), dementia is a clinical syndrome “characterized by the presence of marked impairment in two or more cognitive domains relative to that expected given the individual’s age and general premorbid level of cognitive functioning, which represents a decline from the individual’s previous level of functioning” [8]. The prevalence of malnutrition increases with the severity of dementia [7]. However, the risk of malnutrition is already greater in individuals with early dementia compared with cognitively healthy older adults [9]. 

Considerable evidence exists on the management of malnutrition in the advanced stages of dementia, but there is a scarcity of information on the subject in the early phases of cognitive disorders. The causes and most efficient treatment strategies of malnutrition in individuals with early stages of dementia are different from those with more advanced diseases. This narrative review aimed to clarify the current knowledge on the prevention and management of malnutrition in older adults with early stages of cognitive disorders. First, we focus on the analysis of mechanisms underlying malnutrition in older adults in general and in those with early-stage dementia. Then, we discuss the current knowledge on the prevention and treatment of malnutrition in this patient population. Finally, we review the evidence on the effects of vitamin supplements and specific dietary patterns on preventing cognitive decline. 

## 2. Mechanisms of Malnutrition in Old Age

Age-related changes in the regulation of appetite have a central role in the so-called “anorexia of aging”, defined as the loss of appetite and/or decreased food intake in late life [10]. Several mechanisms are responsible for and mutually interconnected in the decline of appetite with aging, such as olfactory dysfunction, decreased hunger, increased satiety, chronic low-level inflammation, and alterations in gastric motility [10]. The sense of smell and taste deteriorates with aging. Olfactory dysfunction affects up to 60% of individuals over 65 years [11]. Physiological age-related changes in olfactory and gustatory function include a decreased number of taste buds and atrophy of the remaining ones [10], impaired ability to regenerate olfactory neurons [12], reduced saliva secretion, and a decreased release of odor molecules from foods due to impaired chewing [12]. Diseases, medications, smoking, and environmental exposures may worsen these changes [10,11,13]. This may contribute to a reduced nutrient intake and have an influence on food choices, usually resulting in a less varied diet [10].

Aging also influences the levels of several hunger and satiety hormones, such as ghrelin, leptin, insulin, cholecystokinin (CCK), and peptide YY, which contribute to the anorexia of aging [10]. Ghrelin is a hunger-stimulating hormone released during fasting by the gastrointestinal mucosa in the stomach [14]. Its sensitivity decreases because of a concomitant increase in the satiety hormones insulin and leptin [15]. Insulin acts directly as a satiety hormone and indirectly as an enhancer of the anorexigenic stimulus of leptin in the hypothalamus [16,17]. The levels of leptin, produced by the adipose tissue, and insulin, produced by the pancreas, rise in the blood as a long-term signal of adiposity [16]. Blood levels of leptin and insulin correlate with adiposity levels in the adipose tissue’s central system for energy storage and potentiate the central satiety sensation. CCK is a satiety hormone released in response to the delivery of nutrients in the proximal small intestine [18]. Higher circulating levels of leptin and CCK and an increased fasting and post-prandial plasma insulin concentration have been found in older adults compared with younger individuals [17,19,20,21,22,23]. High CCK levels are correlated with greater satiety after meals, thereby contributing to the anorexia of aging [21]. Studies have also shown an increase in circulating concentrations of peptide YY in the late postprandial phase in older adults [24]. Peptide YY, a hormone produced after meals by specialized enteroendocrine cells (L-cells) mainly located in the distal gastrointestinal tract, induces satiety over the short term [25]. Long- and short-term signals of satiety interact so that insulin inhibits ghrelin and leptin reinforces the CCK signal, and vice versa [26]. This process is accompanied by an increase in fasting and postprandial plasma insulin concentrations that, in turn, hinder the orexigenic stimulus of ghrelin in the hypothalamus [17].

Chronic low-grade inflammation, a hallmark of the aging process [27], may modify the response of the hypothalamus to peripheral stimuli [10]. Circulating levels of interleukin (IL)-1, IL-6, and tumor necrosis factor alpha (TNF-α) are generally higher in older adults compared with younger individuals, independent of specific diseases or multimorbidity [28]. TNF-α and IL-1 stimulate leptin mRNA expression, thereby increasing its circulating levels [29]. Inflammation is also associated with delayed gastric emptying and clampdown of small intestinal motility [25] as well as skeletal muscle loss [30,31,32]. Indeed, inflammatory biomolecules interact with prostaglandin and corticotrophin-releasing factor to inhibit gastric emptying [31]. Individuals with chronic obstructive pulmonary disease suffer from both the consequences of energy wasting (due to respiratory inefficiency) and anorexia, caused by inflammatory mediators. Similarly, cancer and heart failure cause cachexia by increasing energy requirements and anorexia. In these conditions, high concentrations of cytokines such as IL-6 and TNF-α have a strong anorexigenic effect [33]. 

In addition to these mechanisms, abnormalities in gastrointestinal function are involved in the anorexia of aging [10]. Altered gastric motility may cause early satiation, which is correlated with reduced fundus compliance. Due to age-related changes, the fundus of the stomach in older adults produces less nitric oxide (NO) [16]. NO is a ubiquitous gaseous molecule that is thought to be the main inhibitory neurotransmitter responsible for gastrointestinal relaxation in response to gastric filling [34]. A lack of NO diminishes gastric compliance and leads to rapid antral filling, giving a sensation of satiety [25]. In addition, delayed gastric emptying may decrease appetite and food intake by enhancing and prolonging antral distension as well as modifying small intestine satiety signals [10]. Moreover, chronic gastritis and some drugs (e.g., proton-pomp inhibitors) may cause hypochlorhydria, which further delays gastric emptying [35]. 

## 3. Risk Factors for Malnutrition in Early Cognitive Disorders

Cognitive, psychological, social, pathophysiological, and medication-related factors increase the risk of malnutrition in individuals with early cognitive disorders.

### 3.1. Cognitive, Psychological, and Social Factors

Mild dementia is characterized by a moderate memory loss interfering with everyday life, moderate difficulties in time and space orientation and problem handling, difficulties in community affairs, a mild impairment of daily functioning, and a need for prompting [36]. Executive function impairment, which influences an individual’s ability to plan, organize, and complete tasks, and attention deficits are common early symptoms of cognitive disorders [37,38]. Thus, adults with early dementia often face challenges and difficulties in the acquisition of food products and food preparation, as well as maintaining their previous food intake [39,40,41,42,43]. Indeed, difficulties in daily functioning are associated with poorer nutritional status in community-dwelling individuals with newly diagnosed Alzheimer’s disease (AD) [44]. In addition, difficulties in maintaining attention while eating are associated with poor appetite in those with mild cognitive impairment (MCI) [45]. 

Changes in appetite, depression, and apathy, which are frequent behavioral and psychiatric symptoms of dementia (BPSD) in the early disease stages, may increase the risk of malnutrition [46,47,48]. The prevalence of depression in individuals with mild dementia is about 38%, and the prevalence of apathy is greater than 50% [49]. Depression is associated with an increased risk of malnutrition, as a decrease in appetite is one of the core clinical features of depression [50]. In addition, apathy has been associated with malnutrition in older women with MCI and early-stage AD [51].

Social support has a key role in the everyday lives of individuals with mild dementia. However, social support is frequently inadequate for these people. This may be caused by a lack of social relationships but also by the fact that those suffering from early dementia often refuse to accept help from other people, even if there are clear deficiencies in their self-care abilities [43]. This is because anosognosia, an inability to recognize cognitive, behavioral, and functional impairment caused by a dementing illness, is a common symptom of mild dementia [52]. A lack of support may contribute to the development of malnutrition.

### 3.2. Pathophysiological Factors

Pathophysiological changes associated with the early stages of dementia, such as olfactory and gustatory dysfunction, increase the risk of malnutrition. As mentioned earlier, olfactory and gustatory functions decline with age, but their deterioration is accelerated in the preclinical stages of dementia [53,54,55,56]. The basis of olfactory dysfunction in the setting of cognitive disorders is not well established, but it is suggested that cortical atrophy and gray matter abnormalities in the brain related to cognitive aging and cognitive disorders may play a role [57]. Unexpectedly, many studies did not find an association between olfactory dysfunction and malnutrition in older adults [12,58,59,60]. Notwithstanding, Kim et al. [61] showed that older adults with a high smell threshold had a lower intake of calories, protein, fat, carbohydrates, and minerals than those with a lower threshold. 

Involuntary weight loss is common in individuals with mild dementia and even in preclinical disease stages [62,63,64,65]. The mechanisms behind weight loss are only partially understood. While weight loss indicates a negative energy balance, it is unclear if this is due to a decreased food intake and/or an increased energy expenditure. A systematic review included seven articles that compared energy and protein intake between individuals with AD and cognitively healthy controls [66]. The results indicated no differences in food intake, but the heterogeneity of findings was high, and study methodologies were considered to be of poor or moderate quality. Most studies considered patients with moderate or severe dementia, and only one study included individuals with mild dementia [67]. The latter found that the intake of nutrients and energy was significantly lower in individuals with mild dementia than in cognitively healthy older adults [67]. In contrast, Doorduijn et al. [68] did not find differences in food intake, but they showed that resting energy expenditure was increased in individuals with MCI and early AD compared with controls. A recent systematic review by Porter et al. [69] included six studies that compared total energy expenditure in people with and without dementia. The results showed that there were no significant differences in total energy expenditure between these groups. However, the included studies included dementia patients as a single group and did not account for disease severity. Hence, additional studies are needed to clarify the reasons for involuntary weight loss in individuals with preclinical and early dementia stages.

### 3.3. Medication-Related Factors

One of the pathophysiological mechanisms of AD is a decrease in neurotransmitter acetylcholine (ACh) levels in the brain. The cerebral availability of ACh can be increased pharmacologically by blocking the activity of the acetylcholinesterase enzyme (AChE), which is responsible for the degradation of ACh. AChE inhibitors, namely donepezil, galantamine, and rivastigmine, are the front-line pharmacotherapies in the treatment of cognitive and behavioral symptoms in mild and moderate AD [70]. Because AChE inhibitors increase cholinergic activity not only in the brain but also in the gastrointestinal system, common side effects, particularly at the beginning of treatment, include nausea, vomiting, and diarrhea. Soysal et al. [71] conducted a systematic review and meta-analysis exploring the influence of therapy with AChE inhibitors on weight change. The meta-analysis of nine randomized controlled trials (RCTs) showed a twofold increased risk of weight loss in participants on AChE inhibitors compared with those taking placebo. In addition, a significant cumulative incidence of weight loss was observed in longitudinal and open-label studies in patients taking AChE inhibitors. Thus, the use of AchE inhibitors may increase the risk of malnutrition.

## 4. Consequences of Malnutrition

### 4.1. Adverse Health Outcomes of Energy-Protein Malnutrition

**Figure 1:**
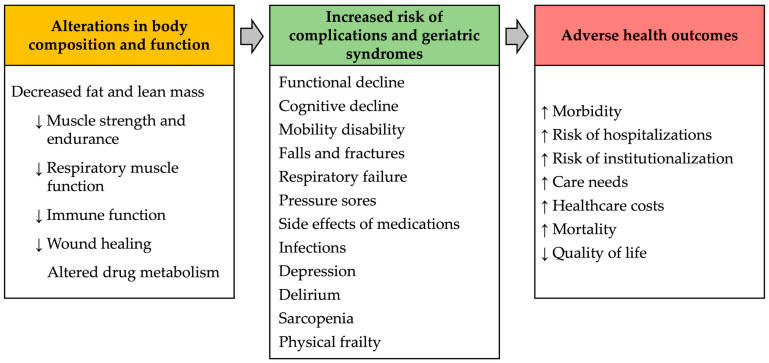
Consequences of malnutrition in older adults.

Energy-protein malnutrition causes alterations in body composition and function that, in turn, increase the risk of complications and predispose to serious adverse health outcomes (Figure 1) [3]. Sarcopenia is a common condition in older adults that is associated with malnutrition [72] and cognitive impairment [73]. Together, these geriatric syndromes cause a vicious circle that worsens the severity and outcomes of each of them [39]. 

In cross-sectional studies, malnutrition has been associated with cognitive impairment and dementia [74,75], as well as with BPSD in individuals with mild dementia [51]. Longitudinal studies in patients with dementia examined the associations between malnutrition and rates of cognitive and/or functional decline. Borda et al. [76] followed 202 patients diagnosed with mild dementia for five years. Their results showed that malnutrition at baseline and over the follow-up was a significant predictor of functional decline but not of cognitive decline. In contrast, in the Cache County Dementia Progression Study [77], which followed 292 patients with dementia for six years, malnutrition predicted faster rates of cognitive decline. Kishino et al. [78] showed that a poor nutritional status was associated with worsening of BPSD, especially verbal aggressiveness and emotional disinhibition, over a 2.5-year follow-up in women with MCI and AD.

### 4.2. Micronutrient Deficiencies

#### 4.2.1. B-Complex Vitamins

B-complex vitamins are a group of eight water-soluble vitamins that have essential and closely interrelated roles in cellular metabolism [79,80]. They act as co-enzymes in various enzymatic reactions and have critical roles in the biosynthesis of RNA and DNA. In particular, B vitamins have essential roles in cellular energy production within mitochondria, where they act as co-enzymes in the citric acid cycle and the electron transport chain, as well as in the metabolism of glucose, fatty acids, and amino acids. B vitamins are also essential for the synthesis of several neurochemicals, signaling molecules, and other substrates in the folate and methionine cycles [79]. Hence, normal brain function is dependent on the continuous supply of B-complex vitamins [79]. Most of them are not stored in the body and, therefore, require regular intake to avoid deficiency. The exception is vitamin B_12_, which is stored in the liver, kidney, and other body tissues [80,81]. For this reason, vitamin B_12_ deficiency is usually manifested only after a few years of insufficient intake. However, if the reason for its deficiency is malabsorption, the symptoms and consequences of vitamin B_12_ deficiency become apparent more rapidly [81]. 

There are several reasons that contribute to susceptibility to B vitamin deficiencies in old age. First, the intake of foods that are sources of B vitamins tends to decrease with aging [82]. This may lead to an inadequate vitamin intake and, therefore, contribute to the development of vitamin deficiency. Second, the prevalence of chronic diseases that alter the absorption of B vitamins is increased in older adults. An example of this is atrophic gastritis, which causes malabsorption of vitamin B_12_, leading to its deficiency if not adequately supplemented. The main causes of atrophic gastritis include chronic *Helicobacter pylori* infection, autoimmune gastritis, and the long-term use of medications that lower the acidity of the stomach, like proton pump inhibitors and H2-receptor antagonists [83]. Third, many medications that are commonly used by older adults have an influence on the absorption or metabolism of B vitamins [80,84]. Examples of these are proton-pump inhibitors, H2-receptor antagonists, and metformin that cause malabsorption of vitamin B_12_ [85].

Vitamins B_1_ and B_12_ are the most studied B-complex vitamins in the context of cognitive disorders, and their deficiencies are clearly associated with cognitive impairment and dementia [86,87]. However, any B vitamin deficiency has been shown to disrupt normal brain function and cause cognitive and/or neuropsychiatric symptoms (Table 1). 

#### 4.2.2. Vitamin D

The main reasons for vitamin D deficiency, commonly defined as serum/plasma 25-hydroxyvitamin D levels below 25–30 nmol/L [93], include a lack of exposure to sufficient UVB light and poor nutritional intake [94]. It should be noted that few foods naturally contain vitamin D. These include oily fish (salmon, mackerel, and herring), mushrooms exposed to sunlight or sundried, cod liver oil, and meat such as pork and beef [95,96,97]. As for sunlight, exposure to the sun above and below approximately 33° latitude in the winter does not result in any significant production of vitamin D [98,99]. There are also other factors involved in vitamin D deficiency. Single nucleotide polymorphisms in genes related to vitamin D metabolism are known to affect the level of 25-hydroxyvitamin D and the response to supplementation [100]. Inflammation has also been shown to reduce 25-hydroxyvitamin D [93], and some studies found increased vitamin clearance in association with conditions such as hyperparathyroidism [101] and low calcium intake [102]. 

Vitamin D deficiency has been associated with cognitive decline in cross-sectional and longitudinal studies. Systematic reviews and meta-analyses by Chai et al. [103], Goodwill and Szoeke [104], van der Schaft et al. [105], Sommer et al. [106], and Shen and Ji [107] confirmed significant associations between vitamin D deficiency and dementia and showed that the risk of dementia increased with the severity of vitamin D deficiency. 

## 5. Prevention and Management of Malnutrition

In the following paragraphs, we describe strategies for malnutrition prevention and treatment in older adults, with a special focus on the aspects that are important to consider in individuals with early stages of cognitive disorders.

### 5.1. Screening and Assessment of Malnutrition

**Figure 2:**
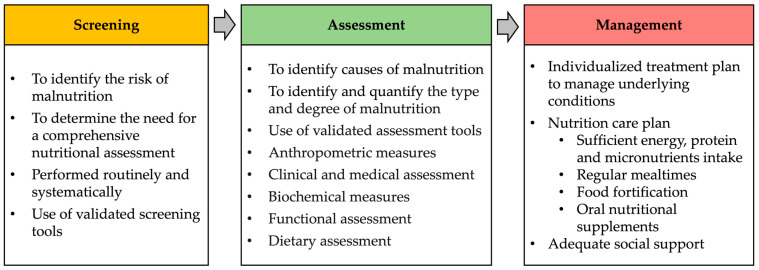
Screening, assessment, and management of malnutrition in older adults.

Routine screening for malnutrition is imperative for its prevention and the recognition of individuals who are either at risk of malnutrition or already malnourished (Figure 2) [108]. 

Even though malnutrition is widespread in older adults with dementia and is associated with poor outcomes, it easily remains undetected unless systematically screened. Therefore, ESPEN recommends that every person with dementia be routinely screened for malnutrition using validated tools [39]. The consensus statements of most clinical nutrition societies (including ESPEN) recommend that the diagnosis of malnutrition in older adults is based on a two-step process (screening and assessment) and Global Leadership Initiative on Malnutrition (GLIM) criteria [109]. GLIM criteria comprise three phenotypic criteria (low body mass index (BMI), involuntary weight loss > 5% in the last six months, and reduced muscle mass) and two etiological criteria (reduced food intake or assimilation of nutrients and inflammation or disease burden). In order to diagnose malnutrition, at least one phenotypic criterion and one etiological criterion should be present. The severity of malnutrition is based on phenotypic criteria. 

The Mini Nutrition Assessment–Short Form (MNA–SF) is a frequently used screening tool in older adults and is suitable for those with dementia [110]. In the latter case, to reduce misjudgment, it is recommended that the patient’s answers be confirmed by relatives or caregivers. The MNA–SF includes items about food intake, weight loss, mobility, acute conditions, neuropsychological problems, and BMI. The resulting score differentiates individuals as having normal nutritional status, being at risk of malnutrition, or being malnourished. Given the high prevalence of weight loss in patients with early stages of dementia, regular monitoring of weight is another useful method for the early detection of malnutrition risk.

If the screening reveals an increased risk of malnutrition or malnourishment, it is necessary to perform a comprehensive assessment of the patient’s nutritional, medical, and functional status to identify underlying causes of malnutrition and quantify its type and degree [108] (Figure 2). It is also necessary to perform a dietary assessment to obtain information on the patient’s usual food and beverage intake. Validated nutrition assessment tools are recommended to be used to support assessment. The most commonly used is the MNA (complete form), which comprehensively evaluates the nutritional status and causes of malnutrition [111]. 

There are usually many simultaneous underlying causes of malnutrition. Common treatable causes include mastication and swallowing problems, side effects of medications, depression, loneliness, pain, anxiety, restricted mobility, constipation, difficulties in food preparation or acquisition, and social problems (lack of support, family conflict) [112,113]. In the medical evaluation, it is also imperative to rule out micronutrient deficiencies that may be a cause of or at least take part in the process of cognitive impairment.

### 5.2. Management of Malnutrition

#### 5.2.1. Individualized Management of Malnutrition

The management of malnutrition is based on the findings of the assessment. It includes the treatment or management of identified underlying causes of malnutrition and the implementation of nutritional strategies to improve energy, protein, and micronutrient intake (Figure 2). 

#### 5.2.2. Strategies to Ensure Adequate Food Intake

Nutritional strategies to improve food intake include guidance on regular mealtimes and avoidance of long fasting periods during the day and night, food fortification, oral nutritional supplements (ONS), and appealing and appetizing foods according to personal preferences. Dietary restrictions are not recommended, as they may increase the risk of malnutrition. Food fortification is useful to support adequate dietary intake. Several systematic literature reviews evaluated the effects of dietary enrichment with conventional foods and showed an increase in protein and energy intake using food fortification [114,115,116].

ONS are products that contain a combination of macro- and micronutrients designed to improve dietary intake when diet alone is insufficient to reach nutritional goals [117]. ONS are used in addition to a balanced diet. Studies have shown positive effects of ONS on body weight and BMI in individuals with dementia [118,119].

Nutrition-related problems such as eating behavior disorders associated with dementia pose a heavy burden on caregivers, who may become stressed, depressed, and socially isolated. Caregiver stress, in turn, is associated with malnutrition of older adults being cared for [120], and it may also aggravate nutritional problems in individuals with dementia. Nutritional education programs designed for caregivers, including information on weight loss, nutritional needs, and interaction during mealtimes, may reduce the stress of the caregiver. Furthermore, as male caregivers often have poor knowledge of cooking and other household activity skills, they could benefit from specific training considering these skills [121]. Scientific evidence has shown a positive effect of caregiver education on the nutritional status of older adults with dementia [122,123]. In addition, modifying environmental factors (like sounds and lightning) to ensure a pleasant environment while eating may improve the nutritional status of individuals with dementia [124,125]. It is also demonstrated that eating in company stimulates dietary intake [124,126,127]. Mealtime interventions with a focus on the social elements of eating and drinking have been shown to increase energy intake in older adults [128].

Appetite stimulants (orexigenic drugs) are an available method to support food intake in patients with a loss of appetite. However, there is a lack of studies on their use in individuals with dementia. Because these drugs have various side effects, they are not generally recommended in this setting [129,130].

#### 5.2.3. Prevention of Dehydration

The central nervous system consists of 80% water, stored in astrocytes [131]. Aging causes alterations in water homeostasis through several mechanisms (e.g., renal function alterations, regulation of thirst, vasopressin secretion changes, alterations of body composition) that increase the risk of dehydration and hyperosmolality [132]. Chronic hypovolemia, due to long-lasting hypohydration, may reduce brain volume and disrupt normal neural pathways, thus predisposing older adults to cognitive decline [131,133]. Lauriola et al. [134] showed that hypovolemia was more prevalent in individuals with cognitive impairment and dementia compared with cognitively healthy older adults. Dehydration was also associated with lower MNA scores. The PREDIMED-Plus study included 1957 individuals with metabolic syndrome [135]. More than half of the participants were physiologically dehydrated. Lower hydration status was associated with a greater decline in global cognitive function over a 2-year follow-up. The Berlin Aging Study showed that chronic dehydration was associated with a steeper decline in cognitive functioning over time in older adults [136]. These results highlight the importance of preventing, recognizing, and treating fluid balance disorders at an advanced age.

#### 5.2.4. Replacement Therapy for Vitamin Deficiency and Cognition

Considering the essential roles of vitamins B and D in the physiological functions of the body, replacement therapy for their deficiencies is beneficial for the general health of patients. There is also evidence that, if replacement therapy for vitamin B deficiency is started before irreversible damage to the central nervous system has occurred, cognitive functions may be restored [137,138,139,140,141] (Table 2). However, if dementia has already developed, cognitive impairment is most probably irreversible. The findings on the effects of replacement therapy for vitamin D deficiency on cognition are mixed; some studies found a significant beneficial effect on cognitive functions [142,143,144,145], while others did not confirm these findings [146,147,148] (Table 2).

## 6. Evidence on Nutritional Strategies Aimed at Preventing Cognitive Decline

### 6.1. Vitamin Supplements 

Systematic reviews and meta-analyses have summarized the evidence on RCTs that have examined the effects of vitamin B or D supplements on improving cognitive functions or preventing cognitive decline in older individuals without vitamin deficiencies (Table 3) [149,150,151,152,153,154,155,156,157,158,159,160,161]. These studies show that there are no beneficial effects of vitamin B or D supplements on cognitive functions, and they are therefore not recommended to improve cognition or to prevent cognitive decline in either cognitively healthy older adults or in those with dementia.

### 6.2. Healthy Dietary Patterns for Neuroprotection

There is an increasing interest in healthy dietary patterns and specific diets that may prevent cognitive decline [162,163]. A meta-analysis of observational studies by Liu et al. [164] and a systematic review by van de Rest et al. [165] found that adherence to high-quality diets or healthy dietary patterns was associated with a lower risk of cognitive impairment compared with participants on low diet quality or an unhealthy dietary pattern.

The Mediterranean diet (MeDi) is an extensively studied dietary pattern with cardiovascular health benefits yielded by a high consumption of fruits, vegetables, legumes, whole grains, and olive oil, moderate consumption of fish, and a low consumption of red meat and foods containing saturated fats [166]. The PREDIMED trial enrolled 447 participants without cognitive impairment (233 women; mean age, 66.9 years) and compared the effects of three different dietary patterns on cognitive functions: MeDi supplemented with extra virgin olive oil (1 l/week), MeDi supplemented with mixed nuts (30 g/die), and a control diet [167]. The adoption of MeDi enriched with olive oil or nuts for 4 years was associated with better cognitive functions compared with the control group. A meta-analysis by Coelho-Junior et al. [168] found that high MeDi adherence was associated with better global cognition and a reduced risk of decline in global cognitive function, but no benefit was observed for the prevention of cognitive impairment or dementia. Similar findings were reported in a systematic review by Limongi et al. [169]. A systematic review by Petersson and Philippou [170] concluded that adherence to a MeDi was associated with better cognitive performance in cross-sectional studies, but the causal relationship remained unclear. A systematic review by Lourida et al. [171] showed that higher MeDi adherence was associated with better cognitive function, lower rates of cognitive decline, and a reduced risk of AD. Singh et al. [172] included only prospective studies in their meta-analysis and concluded that a higher adherence to a MeDi was associated with a reduced risk of developing cognitive impairment and of progressing from MCI to AD. However, a systematic review by Radd-Vagenas et al. [173], including only RCTs, did not find beneficial effects of MeDi on cognition. More longitudinal studies with long-term follow-up are needed to evaluate the effectiveness of MeDi in the prevention of dementia.

The Dietary Approaches to Stop Hypertension (DASH) diet [173] and the MeDi–DASH Diet Intervention for Neurological Delay (MIND) [174] are dietary patterns based on MeDi that have been further developed to achieve health benefits. The DASH diet is designed to prevent and treat hypertension and is based on a high consumption of plant-based foods and a low intake of total fat, saturated fatty acids, cholesterol, and sodium [173]. The MIND dietary pattern has been developed to prevent dementia, and it is a combination of the MeDi and DASH diets, enriched by foods with neuroprotective properties like berries and green leafy vegetables [174]. van den Brink et al. [174] compared the effects of MeDi, DASH, and MIND diets on cognitive functions in middle-aged and older adults. The authors concluded that all these dietary patterns were associated with a lower risk of cognitive decline compared with control groups, with the strongest associations reported for the MIND diet. Similarly, a systematic review by Solfrizzi et al. [175] found that higher MeDi adherence was associated with better cognitive functions and that the DASH and MIND diets were associated with slower rates of cognitive decline and a reduction in AD rates compared with control diets. More studies are needed to certify the benefits of the MIND and DASH diets for the prevention of dementia.

## 7. Conclusions

Individuals with early stages of cognitive disorders are susceptible to energy-protein malnutrition and micronutrient deficiencies owing to factors related to biological aging and special features of cognitive disorders that influence food intake and energy expenditure. To avoid the adverse health outcomes of malnutrition, prevention strategies should be systematically implemented in the care of individuals with early dementia. These include routine screening for malnutrition, regular monitoring of body weight, and the provision of adequate support in everyday life. If malnutrition or a condition of risk is detected, a comprehensive assessment of the individual’s situation should be undertaken, and a personalized plan for the management of malnutrition and the treatment of underlying causes should be developed. Micronutrient deficiencies should be promptly identified and treated. There is evidence that early treatment of B and D vitamin deficiency may restore cognitive decline due to vitamin deficiency. However, if dementia has already developed, the changes in cognitive function are most likely irreversible. In contrast, strong evidence exists against the use of vitamin B and D supplements for the prevention of cognitive decline in individuals without deficiency. Instead, adherence to healthy dietary patterns may have predictive effects on cognitive decline. For example, MeDi is associated with better cognitive performance in cross-sectional studies. Some longitudinal studies have indicated that healthy diets may prevent cognitive decline and diminish dementia rates, but more longitudinal studies with longer follow-up and clinically meaningful outcome measures are needed to confirm these findings.

## Figures and Tables

**Table 1:** B complex vitamins, risk factors for their deficiencies, and cognitive and neuropsychiatric symptoms of their deficiencies in adults.

Vitamin		Source	Risk Factors for Deficiency	Cognitive and Neuropsychiatric Syndromes of Deficiency	Ref.
B_1_	Thiamin	Plant and animal products	Poor intake (alcohol abuse, prolonged fasting, malnutrition), malabsorption (gastrointestinal malignancies, upper gastrointestinal or pancreatic surgery), increased utilization (hyperthyroidism), increased loss (diarrhea, advanced kidney disease, dialysis), drug-nutrient interaction (diuretics)	Wernicke–Korsakoff syndrome, cognitive decline, depression	[79,80,86,88]
B_2_	Riboflavin		Poor intake (alcohol abuse, malnutrition, vegan diet), malabsorption (diabetes, liver disease, thyroid, and renal insufficiency), increased loss (diarrhea, dialysis)	Brain dysfunction, personality change	[79,80,89,90]
B_3_	Niacin		Poor intake (alcohol abuse, malnutrition), malabsorption (Crohn’s disease), drug-nutrient interaction (azathioprine, 6-mercaptopurine, 5-fluorouracil)	Depression, anxiety, cognitive decline, psychotic symptoms	[79,80,91]
B_5_	Pantothenic acid		Poor intake (alcohol abuse), malabsorption (diabetes, inflammatory bowel diseases), drug-nutrient interaction (cholinesterase inhibitors, memantine)	Cognitive impairment, depression	[79,80,92]
B_6_	Pyridoxine		Poor intake (alcohol abuse, malnutrition), malabsorption (advanced kidney disease), drug-nutrient interaction (steroids, valproic acid, carbamazepine, phenytoin, metformin)	Irritability, impaired alertness, depression, cognitive decline, dementia	[79,80,85,90]
B_7_	Biotin		Poor intake (alcohol abuse), malabsorption (genetic disorders), drug-nutrient interaction (anticonvulsants)	Depression	[79,80,84]
B_9_	Folic acid/folate	Plant products	Poor intake (alcohol abuse, malnutrition); malabsorption (intestinal diseases); others (low B_2_ and B_12_ levels); drug-nutrient interaction (methotrexate, metformin, cholestyramine, anticonvulsants)	Affective disorders, behavior changes, psychosis, cognitive decline, dementia	[79,80,87,90]
B_12_	Cobalamin	Animal products	Poor intake (alcohol abuse, vegetarian diet, malnutrition), malabsorption (intestinal diseases, gastric/intestinal resection, atrophic gastritis, bacterial overgrowth, Helicobacter pylori, pancreatic insufficiency), drug-nutrient interaction (proton-pump inhibitors, H2-receptor antagonists, metformin)		

**Table 2:** Original studies on the effects of vitamin B or D replacement therapy on cognitive functions.

Vitamin	Study Design and Population	Intervention	Results	Ref.
B_12_	Longitudinal case-control study: 88 patients with B_12_ vitamin deficiency; *n* = 66 with dementia; *n* = 22 with cognitive impairment	Oral B_12_ vitamin supplementation was administered to all participants. A neuropsychological test battery was performed at baseline and after 8 months.	B_12_ supplementation improved cognition in patients with cognitive impairment, but there was no effect in those with dementia or on the deterioration rate of cognition compared with matched controls.	Eastley et al. [137]
	Longitudinal study: *n* = 39 patients with B_12_ vitamin deficiency and cognitive impairment	Oral B_12_ vitamin supplementation was administered to all participants. MMSE was performed at baseline and at follow-up visits (21–133 days).	The mean MMSE score of patients improved significantly from a score of 20.5 (SD 6.4) to 22.9 (SD 5.5).	Ueno et al. [138]
	RCT: *n* = 271 patients with borderline low serum vitamin B_12_ levels and diabetes without cognitive impairment	Patients were assigned to oral B_12_ vitamin supplementation or placebo for 27 months. MMSE and CDR were performed at baseline and at 9-month intervals up to 27 months.	Vitamin B_12_ supplementation did not prevent cognitive decline.	Kwok et al. [139]
B_12_ and B_9_	RCT: *n* = 193 patients with mild vitamin B_12_ deficiency; *n* = 110 without cognitive impairment; *n* = 51 with MCI; *n* = 23 with dementia	Patients were assigned to receive oral vitamin B_12_ supplementation with or without vitamin B_9_; or a placebo for 24 weeks. A neuropsychological test battery was performed at baseline and at week 24.	There was no beneficial effect of vitamin B_12_ supplementation alone or in combination with vitamin B_9_ on cognitive functions (no stratification based on levels of cognitive function impairment was performed).	Eussen et al. [140]
B_9_	RCT: *n* = 30 patients with vitamin B_9_ deficiency and MCI	Patients were assigned to receive oral vitamin B_9_ supplementation or placebo for 60 days. The Randt Memory Test was performed at baseline and at 60 days.	Patients treated with vitamin B_9_ showed a significant improvement in memory and attention efficiency when compared with the placebo group. The degree of memory improvement was positively correlated with the initial severity of B_9_ deficiency.	Fioravanti et al. [141]
D_3_	RCT: *n* = 40 older women with D vitamin deficiency	Participants were assigned to D_3_ and calcium-fortified yogurts or placebo for 3 months. A cognitive test battery was performed at baseline and at 3 months.	The intervention group performed better on the MMSE, but there were no differences in other cognitive test results (TMT, FAB, digit span, and Stroop test).	Beauchet et al. [142]
	RCT: *n* = 210 patients with low serum 25OHD and AD	Patients were assigned to receive oral vitamin D_3_ treatment or a placebo for 12 months. A neuropsychological test battery was performed at baseline, 6 months, and 12 months.	Both patients with AD and MCI who were treated with vitamin D_3_ showed a significant improvement in several cognitive tests compared with controls.	Jia et al. [144]
	RCT: *n* = 163 patients with low serum 25OHD and MCI			Hu et al. [143]
	RCT: *n* = 374 patients with vitamin D deficiency	Patients were assigned to receive vitamin D_3_ treatment or a placebo. The Digit Symbol-Coding Test was performed at baseline and at 4 months.	At the end of the study, there were no statistically significant differences between the two groups in changes in cognitive test scores.	Jorde et al. [146]
	RCT: *n* = 95 critically ill patients with vitamin D deficiency	Patients were assigned to receive enteral vitamin D_3_ treatment or placebo at baseline. Cognition was assessed using the RBANS 1 to 1.5 years later.	No evidence was found that early administration of high-dose (540,000 IU) enteral vitamin D3 improved long-term cognition or executive function in critically ill patients with vitamin D deficiency.	Han et al. [147]
	RCT: *n* = 42 postmenopausal women with slightly low serum 25OHD	Participants were assigned to receive 600 (control group), 2000, or 4000 IU per day of total vitamin D_3_ supplementation for 1 year. Cognition was assessed using the CANTAB only at the end of the trial.	Participants taking vitamin D at 2000 IU per day performed better on learning and memory tests than the other groups.	Castle et al. [145]
	RCT: *n* = 260 postmenopausal women with low serum 25OHD	Participants were assigned to receive vitamin D combined with calcium or a placebo with calcium. MMSE was administered every 6 months.	There was no difference in cognition over time between women taking vitamin D_3_ supplementation and those taking placebo.	Owusu et al. [148]

Abbreviations: AD, Alzheimer’s disease; CANTAB, Cambridge Neurological Test Automated Battery; CDR, Clinical Dementia Rating; FAB, Frontal Assessment Battery; MCI, mild cognitive impairment; MMSE, Mini Mental State Examination; RCT, randomized controlled trial; SD, standard deviation; TMT, Trial Making Test; 25OHD, 25-hydroxyvitamin D.

**Table 3:** Systematic reviews and/or meta-analyses of the effects of B or D vitamin supplements on improving cognition function or preventing cognitive decline.

Vitamin	No. of RCTs	Participants	Results	Ref.
B_6_, B_12_, and B_9_	16	Adults with and without dementia; *n* = 6276	No effects of B_12_ vitamin supplementation alone or in combination with B_6_ and/or B_9_ vitamins were found on any subdomain of cognitive function.	Markun et al. [161]
	14	Adults with and without dementia; *n* = 866	No evidence of the effect of vitamin B_6_, B_12_, and B_9_ supplementations, alone or in combinations, on cognitive functions.	Balk et al. [149]
	31	Adults with and without dementia; *n* = 17,029	Vitamin B_6_, B_12_, and B_9_ supplementation did not show an improvement in cognitive functions.	Ford et al. [150]
	5	Adults with MCI; *n* = 879	The evidence on overall cognitive function was of very low quality. There was little or no effect of B vitamins taken for 6 to 24 months on cognitive functions.	McCleery et al. [151]
	14	Healthy adults; *n* = 27,882	Vitamin B_6_, B_12_, and B_9_ supplementation had little or no effect on global cognition at any time point up to 5 years.	Rutjes et al. [152]
	20	Healthy adults; *n* = 12,697	There was no overall evidence that oral vitamin B supplementation prevented cognitive decline.	Behrens et al. [153]
B_9_	9	Healthy older adults; *n* = 2358	There was no effect of vitamin B_9_ supplementation on cognitive decline.	Wald et al. [159]
B_6_	2	Healthy older adults; *n* = 117	There was no benefit from vitamin B_6_ supplementation on cognitive functions.	Malouf et al. [154]
B vitamins	21	Adults with and without MCI; *n* = 7571	There was a significant effect of B vitamin supplementation on global cognitive function but not on information processing speed, episodic memory, or executive function.	Li et al. [155]
D_3_	20	Healthy adults; *n* = 6700	The review yielded mixed findings and concluded that there was no effect of vitamin D supplementation on cognitive functions.	Beauchet et al. [160]
	24	Adults with and without dementia; *n* = 7557	Vitamin D supplementation had a small but significant positive effect on cognition. A subgroup analysis indicated that the effect size of vitamin D was stronger in participants with baseline vitamin D deficiency.	Chen et al. [157]
	9	Adults without AD; *n* = 2345	There was no significant difference in cognitive functions between participants supplemented with vitamin D and the comparison groups.	Du et al. [156]
	9	Healthy older adults; *n* = 5588	There were positive changes in verbal memory, learning memory, attention, executive function, and global cognitive function.	Silva et al. [158]

Abbreviations: AD, Alzheimer’s disease; MCI, mild cognitive impairment; RCTs, randomized controlled trials.
